# SENSOR-BASED ASSESSMENT OF FALLS RISK OF THE TIMED UP AND GO IN REAL-WORLD SETTINGS

**DOI:** 10.1093/geroni/igz038.033

**Published:** 2019-11-08

**Authors:** Charlene C Quinn, Barry R Greene, Killian McManus, Stephen J Redmond, Brian Caulfield

**Affiliations:** 1 University of Maryland School of Medicine, Baltimore, Maryland, United States; 2 Kinesis Health Technologies Ltd, Insight Centre for Data Analytics, Dublin, Dublin, Ireland; 3 School of Electrical and Electronic Engineering, Dublin, Dublin, Ireland; 4 Insight Centre for Data Analytics, Dublin, Dublin, Ireland

## Abstract

Falls are the leading cause of older adult injury and cost $50bn annually. New digital technologies can quantitatively measure falls risk. Objective is to report on a validated wearable sensor-based Timed Up and Go (QTUG) assessment detailing 11 measures of falls risk, frailty and mobility impairment in older adults in six countries in 38 clinical and community settings. Second objective is to generate individual targeted falls prevention programs. 14,611 QTUG records from 8,521 participants (63% female) (72.7±10.7 years) available for analysis. QTUG time was 13.9±7.4 s; gait velocity was 101.9±32.5 cm/s. 25.8% of patients reported falling in previous 12 months; 26.2% of patients were at high fall risk. 21.5% not reporting a fall, were high fall risk. Participants had slow walking speed (29.8%); high gait variability (19.8%); problems with transfers (17.5%). Easily captured and interpreted sensor data is useful in a population-based approach to quantify falls risk stratification.

